# Instantaneous simulation of fluids and particles in complex microfluidic devices

**DOI:** 10.1371/journal.pone.0189429

**Published:** 2017-12-21

**Authors:** Junchao Wang, Victor G. J. Rodgers, Philip Brisk, William H. Grover

**Affiliations:** 1 Department of Bioengineering, University of California Riverside, Riverside, CA, United States of America; 2 Department of Computer Science and Engineering, University of California Riverside, Riverside, CA, United States of America; Tsinghua University, CHINA

## Abstract

Microfluidics researchers are increasingly using computer simulation in many different aspects of their research. However, these simulations are often computationally intensive: simulating the behavior of a simple microfluidic chip can take hours to complete on typical computing hardware, and even powerful workstations can lack the computational capabilities needed to simulate more complex chips. This slows the development of new microfluidic chips for new applications. To address this issue, we present a microfluidic simulation method that can simulate the behavior of fluids and particles in some typical microfluidic chips instantaneously (in around one second). Our method decomposes the chip into its primary components: channels and intersections. The behavior of fluid in each channel is determined by leveraging analogies with electronic circuits, and the behavior of fluid and particles in each intersection is determined by querying a database containing nearly 100,000 pre-simulated channel intersections. While constructing this database takes a nontrivial amount of computation time, once built, this database can be queried to determine the behavior of fluids and particles in a given intersection in a fraction of a second. Using this approach, the behavior of a microfluidic chip can be simulated in just one second on a standard laptop computer, without any noticeable degradation in the accuracy of the simulation. While our current technique has some constraints on the designs of the chips it can simulate (namely, T- or cross-shaped intersections, 90 degree channel turns, a fixed channel width, fluid flow rates between 0 and 2 cm/s, and particles with diameters between 1 and 20 microns), we provide several strategies for increasing the range of possible chip designs that can be simulated using our technique. As a proof of concept, we show that our simulation method can instantaneously simulate the paths followed by particles in both simple and complex microfluidic chips, with results that are essentially indistinguishable from simulations that took hours or even days to complete using conventional approaches.

## Introduction

Computer-based simulation is becoming increasingly important in the design of microfluidic devices. Finite element analysis (FEA) software enables researchers to study microfluidic phenomena in their chips [1, 2], optimize existing chip designs [3], and even automate the design of new devices [4]. However, existing simulation tools suffer from several notable drawbacks which have unnecessarily raised the barrier to entry and slowed widespread adoption of these tools in the microfluidics community. First, existing software tools for FEA are complex multi-purpose tools with significant “learning curves” and little customization for microfluidics. Second, the computational requirements of performing the FEA simulation can be prohibitive, leading to exorbitant runtimes. For example, a recent numerical simulation of the acoustic viscous torque effect on the rotation of a single particle took *several days* to complete using a quad-core Intel Xeon X5570 CPU workstation with 48 GB of RAM [5]. This is tremendously costly, especially during early-stage design space exploration, when chip designers need to rapidly consider a large number of variations in chip designs. Fast simulation times could also be particularly beneficial for emerging computer-based tools that automatically design application-specific custom microfluidic chips [4].

This work presents a new technique to dramatically reduce the time required to simulate some common microfluidic chips. Our technique is inspired by *memoization*, a method for solving complex problems by breaking them into pre-solved sub-problems [6]. We first decompose a microfluidic chip into two types of components: *channels* and *channel intersections*. The behavior of fluid in the channels is determined using fluid flow models like the Hagen-Poiseuille equation and simple analogies from electronic circuits like Kirchhoff’s Laws. The behavior of fluid and particles in the intersections is determined by querying a database containing tens of thousands of pre-simulated intersections. By combining intersection simulations retrieved from the database with channel calculations obtained using electrical circuit analogies, we construct a complete simulation of the behavior of fluids and particles in a typical microfluidic chip in around one second on a conventional laptop computer, yielding results that are virtually identical to those obtained in hours or days using conventional software.

## Theory of instantaneous microfluidic simulation

The analogy with electronic circuits has long been used to model fluid flow in microfluidic channels [7, 8]. For example, a voltage difference between two points in an electronic circuit is analogous to a pressure difference Δ*P* between two points in a microfluidic chip; likewise, the electrical current and resistance of a wire are respectively analogous to the fluid flow rate *Q* and hydrodynamic resistance *R*_*h*_ of a microfluidic channel. The flow rate in a channel is proportional to the applied pressure drop and inversely proportional to the hydrodynamic resistance of the channel; this is the microfluidic equivalent of Ohm’s Law:

$$\begin{array}{c} Q=\frac{\Delta P}{R_{h}} \end{array}$$ (1)

Also like electronic resistors, microfluidic channels with hydrodynamic resistances *R*_*h*1_, *R*_*h*2_,…*R*_*hn*_ arranged in series have an equivalent hydrodynamic resistance *R*_*h*_ of

$$\begin{array}{c} R_{h}=R_{h1}+R_{h2}+\cdots +R_{hn} \end{array}$$ (2)

and microfluidic channels with hydrodynamic resistances *R*_*h*1_, *R*_*h*2_,…*R*_*hn*_ arranged in parallel have an equivalent hydrodynamic resistance *R*_*h*_ of

$$\begin{array}{c} \frac{1}{R_{h}}=\frac{1}{R_{h1}}+\frac{1}{R_{h2}}+\cdots +\frac{1}{R_{hn}} \end{array}$$ (3)

For microfluidic chips containing a complex network of microfluidic channels, Kirchhoff’s laws (which are ordinarily used to calculate currents and voltages in electrical circuits) can be used to determine the flow rate and pressure at each point in the chip. Specifically, the fluidic analog of Kirchhoff’s current law predicts that the sum of the flow rates of fluid flowing into a channel intersection equals the sum of flow rates of fluid flowing out of the intersection, and the fluidic analog of Kirchhoff’s voltage law predicts that the sum of all of the pressure drops in a loop of channels equals zero.

The non-negligible viscosity of fluid causes the analogy between electronics and microfluidics to break down in certain cases. For example, while the current-carrying capacity of a wire is simply proportional to its cross-sectional area, the flow-carrying capacity of a microfluidic channel is a complex function of the cross-sectional size *and shape* of the channel, as well as the viscosity of the fluid in the channel. Expressions for calculating or estimating the hydrodynamic resistance *R*_*h*_ of a channel exist for channels with certain cross-sectional shapes. Our technique assumes that channels have a rectangular cross-sectional shape (the most common channel cross section shape in microfluidics) and a hydrodynamic resistance of

$$\begin{array}{c} R_{h}=\frac{12\eta L}{wh^{3}F} \end{array}$$ (4)

where *η* is the viscosity of the fluid, *L* is the length of the channel, *w* is the width of the channel, *h* is the height of the channel, and *F* is the rectangular geometric form factor of the device [9]. For microfluidic devices with channel cross-sectional shapes that cannot be approximated by a rectangle, other expressions can be substituted for Eq 4.

By treating the channels in a microfluidic device as a network of resistors and using Eqs 1–4 and Kirchhoff’s laws, it is possible to calculate the average flow rate in each channel in a microfluidic chip. Alternatively, electronic circuit simulation software such as SPICE [10], PSpice (Cadence Design Systems, Inc., Rochester, NY) or Simulink (MathWorks, Natick, MA) can be used to model the chip as a network of resistors and calculate the current (the average flow rate) in each channel.

This analogy between electronic circuits and microfluidic chips cannot predict the behavior of microfluidic channel *intersections*, where the merging and splitting of different fluid streams in these intersections (and the paths followed by particles in these streams) share no obvious analog in electronic circuits. The behavior of fluids in these intersections is nonetheless very important. For example, merging streams of fluids in intersections can generate new solute concentrations, and changes in streamline width in intersections can be used to sort particles like cells by their size [11–13]. Computational finite element analysis (FEA) software *can* predict the behavior of fluids and particles in channel intersections, but the significant computational time required to simulate the behavior of these intersections slows down the design process. In summary, there is an unmet need for simulation approaches that combine the speed of the electrical-fluidic analogy (for simulating channels) with the accuracy of finite element analysis (for simulating intersections).

**Fig 1:**
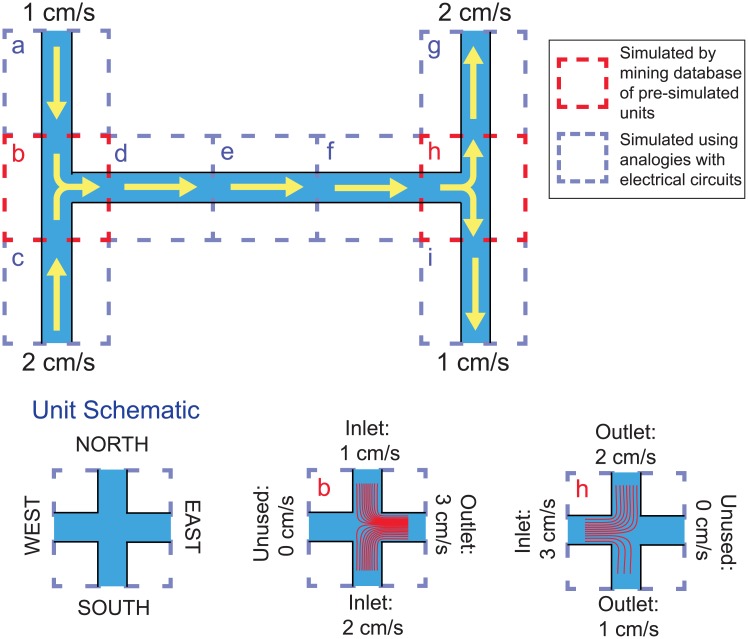
Using instantaneous simulation of a microfluidic chip to predict the paths followed by fluids and particles flowing through the chip. A basic “H” channel chip (top) is first separated into nine units (*a* − *i*). Units *a*, *c*, *d*, *e*, *f*, *g*, and *i* are simple channels; the flow of fluid in these channels is akin to the flow of electricity in a network of resistors and therefore can be modeled using principles of electrical circuit analysis (Eqs 1–3 and Kirchhoff’s laws) and models of hydraulic resistance (Eq 4). Units *b* and *h* are channel intersections where multiple fluid inlets and outlets come together; the paths followed by particles through these intersections cannot be predicted using analogies with electric circuits. Instead, each unit is described in terms of a prototype intersection (the “Unit Intersection,” bottom left) with different boundary conditions. For example, unit *b* has two inlets (1 cm/s at North, and 2 cm/s at South), one outlet (3 cm/s at East), and one unused connection (West). By querying a database containing nearly 100,000 pre-simulated unit intersections, suitable simulation results for units *b* and *h* are retrieved. Particle trajectories from these unit intersections (red lines in *b* and *h*) are then expanded through the rest of the chip using streamline theory [14, 15]. In this manner, the paths followed by fluids and particles through the entire chip are predicted in around one second.

Our proposed solution is to create a database of pre-characterized microfluidic channel intersections. We first introduced a generic Unit Intersection model, shown in Fig 1, which has up to four channels (labeled North, East, South, and West), any of which can be an inlet or an outlet with varying inflow or outflow rates. For example, intersection *b* in Fig 1 has a 1 cm/s inlet at North, a 2 cm/s inlet at South, a 3 cm/s outlet at East, and no connection (*i.e*., a 0 cm/s inlet) at West. We then populated our database with nearly 100,000 different randomly-generated instances of our Unit Intersection, each of which has a different random assignment of Inlets and Outlets and different flow rates at each of the four channels. We then used commercial finite element software to simulate the behavior of fluids and particles in each intersection and stored the results in the database. To determine the behavior of a specific intersection in a given microfluidic chip, one can simply query the database to find the pre-simulated intersection that is the closest match in terms of inlets, outlets, and flow rates, then retrieve that intersection’s simulation results and use them in the overall chip simulation.

Fig 1 shows how our instantaneous microfluidic simulation method is applied to predict the paths followed by particles in a simple “H” channel microfluidic device. Our method first separates the whole chip into nine units (marked *a* − *i*) and then determines whether each unit is a channel or an intersection. Units *a*, *c*, *d*, *e*, *f*, *g*, and *i* are channels, so we can use the electronic-fluidic analogy (Eqs 1–3 and Kirchhoff’s laws) and models of hydraulic resistance (Eq 4) to predict the rate of fluid flow through these units. The remaining two units, *b* and *h*, are channel intersections, so we query our database nearly 100,000 pre-simulated channel intersections to find similar intersections and retrieve their simulation results. Since intersection units *b* and *h* have only three channels, the unused fourth channel in the Unit Intersection is specified to have a flow rate of 0 cm/s. After obtaining fluid streamlines for units *b* and *h* from our database, we then expand these streamlines throughout the rest of the chip based on streamline theory, which predicts that at low Reynolds number, a massless particle will follow fluid streamlines through these channels [14, 15]. In this manner, we can predict the paths followed by fluids and particles in this chip in under one second.

## Materials and methods

**Fig 2:**
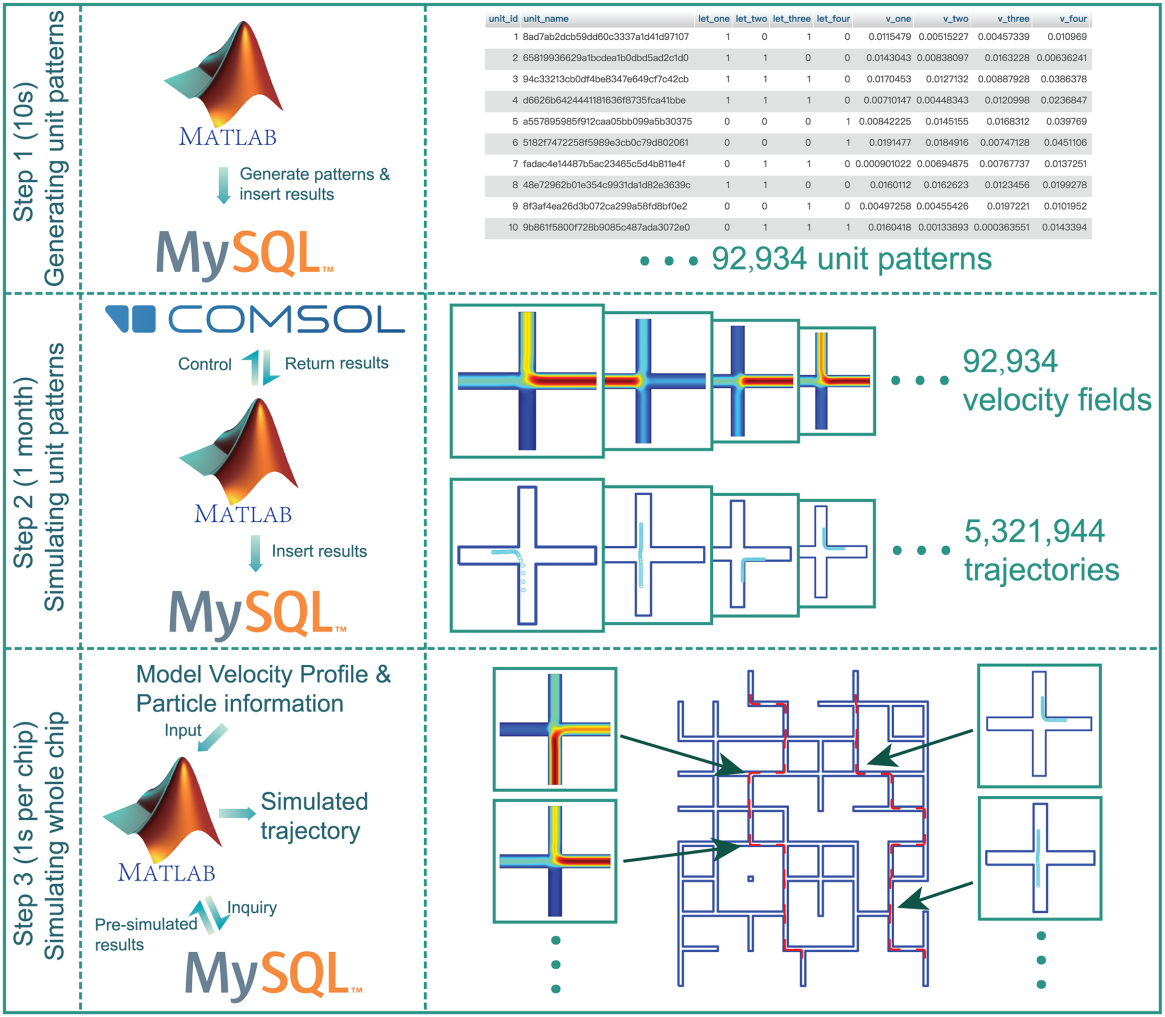
Graphical overview of the instantaneous microfluidic simulation method. In Step 1, MATLAB was used to generate 92,934 unit intersections with different random assignments of inlets and outlets and different random flow rates at each of the four connections North, East, South, and West (see also Fig 1). The resulting unit intersections were generated in 10 seconds and saved into a MySQL database. In Step 2, MATLAB was used to control COMSOL Multiphysics to calculate the fluid velocity fields and particle trajectories of each unit intersection and save the simulation results to the MySQL database. A total of 92,934 fluid velocity fields and 5,321,944 particle trajectories were calculated; the entire simulation process took one month to complete, but this step only needs to be performed once. In Step 3, our method is used to predict the path followed by a particle in a given microfluidic chip. First, the fluid velocity profile and particle information of the chip design are imported into MATLAB. Then MATLAB matches each intersection in the chip design with the closest pre-simulated intersection in the MySQL database and returns the corresponding fluid velocity profile and particle trajectory. Finally, the entire path of the particle through the chip is expanded and generated. Simulating a given chip using this approach takes around one second.

The overall process for instantaneous simulation of microfluidic chips is illustrated Fig 2. We implemented this proof-of-concept demonstration in MATLAB (MathWorks, Natick, MA) using the LiveLink API to automate the simulation of microfluidic intersections in COMSOL Multiphysics and saving device designs and simulation results into a MySQL database [16]. All experimental data was collected on a typical laboratory workstation with a 6-core 3.5 GHz Intel Xeon CPU and 32 GB RAM.

### Step 1: Constructing the database of Unit Intersection instances

Each of the four channels in the generic Unit Intersection shown in Fig 1 can be configured as an inlet or outlet, leading to 16 different possible configurations. The law of conservation of mass (and the fluidic analog of Kirchhoff’s current law) forbids configuring all four channels as inlets or all four channels as outlets, so only 14 of the intersection configurations are actually feasible. When generating random instances of the Unit Intersection, we randomly vary the fluid inflow rates from 0 to 2 cm/s (a typical range of flow rates in microfluidic devices). For an intersection with *N* = 1, 2, or 3 outlets, we randomly vary the outflow rate of *N* − 1 of the outlets from 0 to 2 cm/s. The outflow rate of the remaining outlet is set to be the total inflow rate minus the total outflow rate to ensure conservation of mass. Using this approach, we used MATLAB to generate 92,934 different random instances of the Unit intersection, each of which has a channel width of 200 *μ*m and an overall intersection size of 1.6 × 1.6 mm.

### Step 2: Simulating the behavior of each Unit Intersection instance

The LiveLink API for MATLAB was used to automate the simulation of each of the 92,934 microfluidic intersections in COMSOL Multiphysics. The fluid velocity field for each intersection was solved using the *Laminar Flow* physics module with a customized mesh (1 *μ*m maximum mesh size); we confirmed that finer meshes do not alter the simulation results (thus demonstrating mesh independence). The boundary conditions of the inlets and outlets were defined as described in Step 1 above; the remaining boundaries were specified as walls (no-slip boundary condition) and the material filling the channels was water under incompressible flow. A stationary solver was used for calculation.

We then used the *Particle Tracing for Fluid Flow* physics module in COMSOL Multiphysics to calculate the paths followed by particles through each intersection in our database. A “Drag Force” boundary condition was added to each channel, and a particle “inlet” boundary condition with “Uniform distribution” of initial positions was added to all inlets (10 particles per release). The remaining channels were assigned “Outlet” boundary conditions, and the “Freeze” boundary condition was applied to the walls (meaning that particles in contact with the channel walls will stick there, a realistic assumption in many microfluidic chips). This process was repeated for each intersection using a range of particle diameters from 1 *μ*m to 20 *μ*m. The resulting 5,321,944 simulated particle trajectories were stored in the simulation database. This approach assumes that the entrance lengths of the channels in each intersection (the distance required for a fully-developed flow profile to emerge) is smaller than the lengths of the channels in the simulated intersections.

### Step 3: Simulating the behavior of a given chip

Our code for using our instantaneous technique to simulate a given chip is written entirely in MATLAB; it does not use COMSOL Multiphysics or any other FEA simulation because all necessary FEA results for simulating the chip are available in the MySQL database. The user first specifies which parts of the given chip design are channels and which are intersections (this step will be automated in future versions of our software). The software then loads the fluid velocity profile of the chip, which is used to provide the boundary conditions for each intersection. This fluid velocity profile can be obtained manually (using Eqs 1–4 and Kirchhoff’s laws), using circuit simulation software like SPICE or PSpice, or using computational fluid dynamics simulations obtained from software such as COMSOL Multiphysics. The user then provides the diameter and starting location for each particle to simulate. As simulation proceeds, the software uses streamline theory to project the current position of the particle to the entrance of the next junction. When the software encounters an intersection, it queries the MySQL database to find the pre-characterized unit intersection in the library that most closely matches the boundary conditions of the given intersection. The software retrieves the pre-simulated particle trajectory of the best-matching unit intersection and uses it to predict the path taken by the particle through the intersection. This process repeats until each particle reaches an outlet of the chip, and the software then provides the user with the complete path followed by the particle through the chip. The overall process for simulating the behavior of a given chip takes around one second to complete.

### Comparisons with existing simulation software

**Fig 3:**
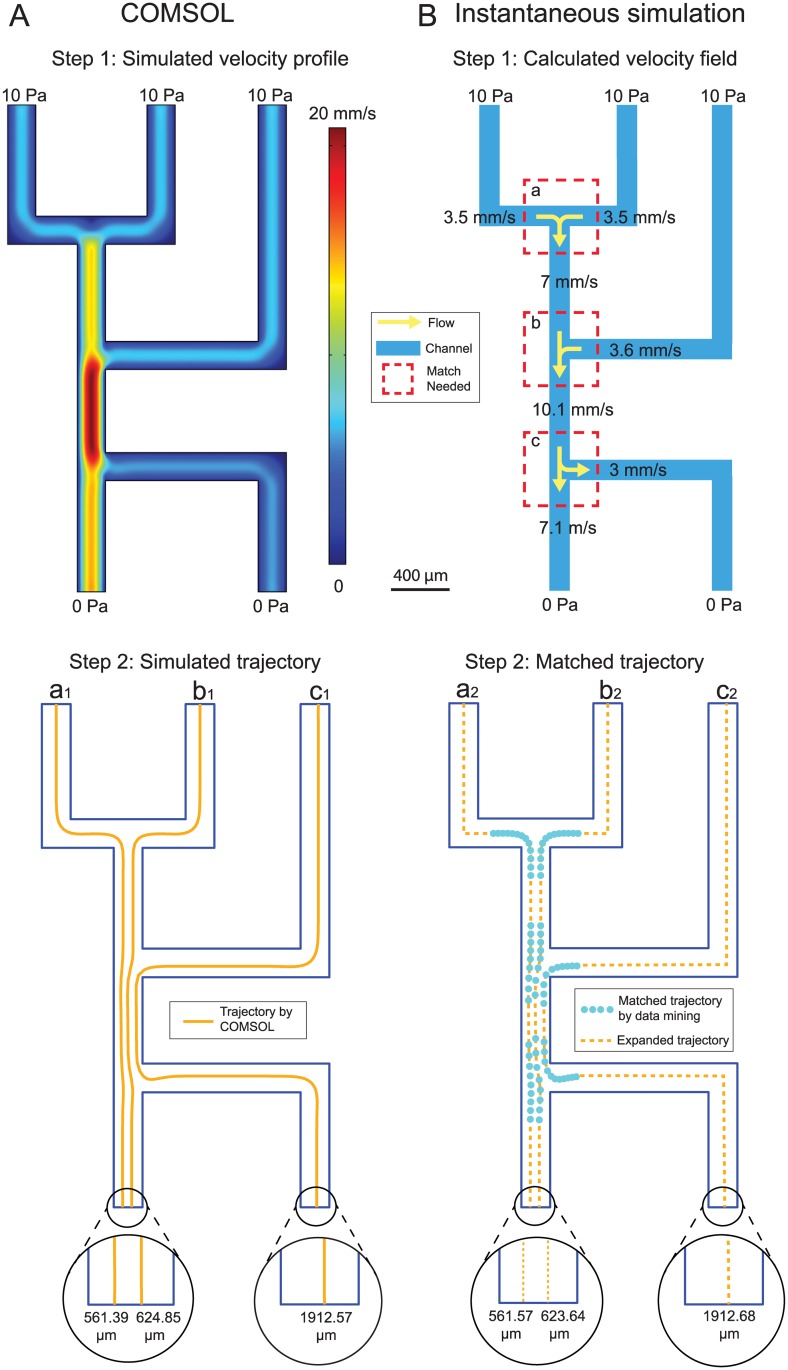
Comparison of results from simulating a simple microfluidic chip design using existing commercial software (COMSOL Multiphysics) and our instantaneous simulation method. In the COMSOL Multiphysics simulation (A), the fluid velocity field is calculated using finite element analysis (Step 1) and the *Particle Tracing for Fluid Flow* physics module is used to calculate the paths followed by particles (Step 2). In our instantaneous simulation (B), the flow rates in the channels were calculated using Eqs 1–4 and fluidic analogs of Kirchhoff’s circuit laws, then simulation results for the channel intersections (dashed red boxes) were found by searching for similar intersections in our database of nearly 100,000 pre-simulated intersections (Step 1). The software retrieved the corresponding particle trajectories from these intersection simulations (blue points in Step 2) and expanded them into whole-chip particle trajectories (yellow points in Step 2). Our instantaneous simulation method was 45 times faster than COMSOL Multiphysics, and the predicted locations of each particle at the exit channels agree to within about 1 *μ*m in a 200 *μ*m wide channel. Raw data of these simulations are available for download (S1 File).

**Fig 4:**
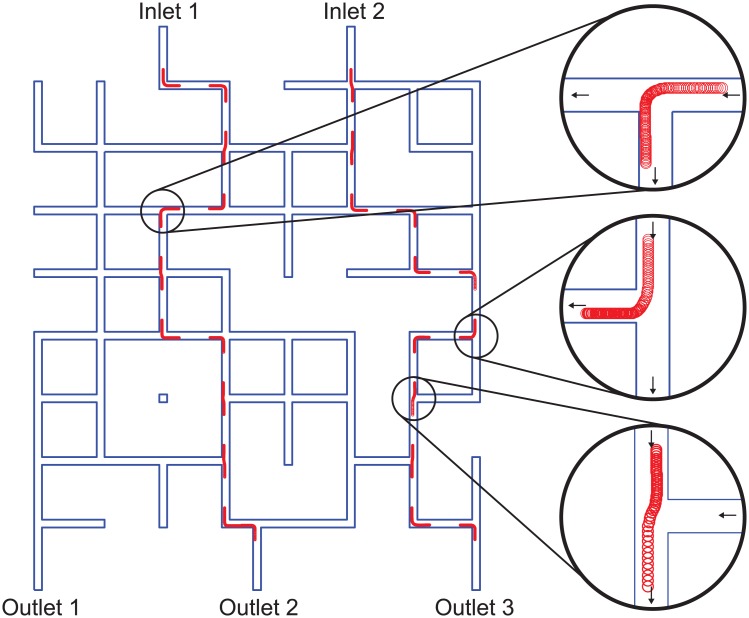
Results from using instantaneous simulation to predict the paths of two 1 *μ*m particles traveling through a randomly-designed microfluidic chip [4], with close-ups of some channel intersections (black circles). The red lines/circles indicate the particle trajectories obtained from the database of pre-simulated intersections, and the gaps between the red lines are regions where no calculation of particle trajectory is necessary because the channels have no intersections in these regions. This simulation was completed in around one second by our instantaneous simulation method. In contrast, the same simulation failed after five days of computation when using an existing simulation tool (COMSOL Multiphysics). Raw data of these simulations are available for download (S1 File).

To compare the computation time and accuracy of the results of our instantaneous simulation method with existing software tools, we also simulated microfluidic chips using only COMSOL Multiphysics. We compared our technique with COMSOL Multiphysics using two chip designs: a simple chip with three inlets and two outlets shown in Fig 3, and a more complex randomly-designed microfluidic chip [4] shown in Fig 4.

For the simple chip in Fig 3, the fluid velocity field of the chip was solved using the *Laminar Flow* physics module in COMSOL Multiphysics and a stationary solver. Each inlet was assigned a boundary condition of 10 Pa absolute pressure and each outlet was assigned a boundary condition of 0 Pa absolute pressure. The simulation used the “Extremely Fine” mesh setting. The *Particle Tracing for Fluid Flow* physics module was then used to predict particle trajectories across the entire chip. A “Drag Force” boundary condition was added to the entire chip, and a particle “Inlet” boundary condition with initial position “Uniform Distribution” and 1.0 *μ*m particle diameter was added to all inlets in the *Laminar Flow* module. The outlets in the *Laminar Flow* module were assigned “Outlet” boundary conditions, and the remaining boundaries were walls (“freeze” boundary condition). The number of particles per release was set to three, meaning that one particle was released in each of the three inlets.

For the randomly designed microfluidic chip in Fig 4, we attempted to solve the fluid velocity field of the chip was again modeled using the *Laminar Flow* physics module in COMSOL Multiphysics as described above, but the simulation failed after five days (details below in *Results and Discussion*). Each inlet was assigned an inlet boundary condition of 0.01 m/s normal inflow rate and each outlet was assigned an outlet boundary condition of 0 Pa absolute pressure. The maximum mesh size was 20 *μ*m.

## Results

Generating the library of 92,934 unit intersection simulations (92,934 fluid velocity fields and 5,321,944 particle trajectories; Step 2 in Fig 2) took approximately one month of continuous computation on a desktop workstation. Each intersection took an average of 27 s to simulate: 5 s to generate mesh coordinates, 2 s to calculate the fluid velocity field, and 20 s to trace the trajectories of particles through the intersection. The resulting database is about 2.1 TB in size (600 MB for the fluid velocity field data, and 1.5 TB for the particle trajectory data). To the best of our knowledge, mesh coordinate generation and particle trajectory calculation in COMSOL Multiphysics is not amenable to parallelization on multiple processors, which limits the performance benefits that can be accrued by using multi-core CPUs in this step. Consequently, the time required to generate the MySQL database depends primarily on CPU clock speed. Memory consumption during library construction did not exceed 4 GB.

Fig 3 presents results using both COMSOL Multiphysics (Fig 3A) and our instantaneous method (Fig 3B) to simulate a simple microfluidic chip with 3 inlets, 2 outlets, and 3 intersections. We simulated the paths followed by 1 *μ*m diameter particles originating at the center of each inlet. The COMSOL-based simulation took 135 s to complete: 15 s to solve the fluid velocity field, and 120 seconds to calculate the particle trajectories. For the instantaneous simulation, Eqs 1–4 and fluidic analogs of Kirchhoff’s laws were used to determine the flow rate boundary conditions of intersections *a*, *b*, and *c*; these boundary conditions are are 3.5 mm/s for the East and West inlets and 7 mm/s for the South outlet of intersection *a*; 7 mm/s for the North inlet, 3.5 mm/s for the East inlet, and 10.1 mm/s for the South outlet of intersection *b*; and 10.1 mm/s for the North inlet, 3 mm/s for the East outlet, and 7.1 mm/s for the South outlet of intersection *c*. We then queried the MySQL database to identify the best matches for each of these three intersections, then expanded the pre-simulated particle trajectories from the database (blue dots) into trajectories that extend through the entire chip (yellow dashes). The entire process of simulating the chip using the instantaneous method took 3 s to complete: 2 s for determining the boundary conditions of the three intersections and obtaining simulation results from the MySQL database, and 1 s to generate the whole-chip particle trajectories. In addition to being 45 times faster than COMSOL Multiphysics, our instantaneous method’s results were virtually identical to COMSOL’s results: the exit locations of the three particles predicted by the two simulations differ by only 0.18, 1.21, and 0.11 *μ*m in a 200 *μ*m wide channel.

To demonstrate instantaneous microfluidic simulation on a more complex chip design, we used the method to predict the paths followed by particles on the large randomly designed microfluidic chip [4] shown in Fig 4. In this simulation, two 1 *μ*m diameter particles start in the center of each inlet channel. The instantaneous simulation method was able to predict the paths followed by the particles in around 1 s of computational time. We attempted to replicate this simulation using COMSOL Multiphysics, but the simulation took *five days* just to generate the coordinates of each mesh node in this design; the subsequent calculation of the fluid velocity field failed, so we were unable to calculate particle trajectories using COMSOL alone.

## Conclusions

We have presented a method to instantaneously simulate the behavior of some common microfluidic chips using conventional computing hardware. The efficiency of our method arises from two key innovations: leveraging existing electrical-fluidic analogies to efficiently compute fluid velocity wherever possible, and querying a database of pre-simulated intersections when necessary. By enabling researchers to simulate microfluidic chip designs in seconds instead of hours or days, our simulation method should accelerate the development of new microfluidic chips.

Our technique bears some similarity to principal component analysis or PCA (also known as proper orthogonal decomposition or POD) in that both our technique and PCA/POD break a complex computation into simpler parts. PCA/POD converts high-dimensional data into lower-dimensional representations (the principal components) that still capture the phenomena of interest [17]. This approach has been applied to a wide range of fluid mechanics problems, including analyzing turbulent flows [18–23] and solving turbulent incompressible Navier-Stokes equations [24]. More recently, Walton *et al*. combined PCA/POD and radial basis functions to solve parameterized fluid flow problems [25], and San *et al*. investigated the PCA/POD closure models in numerical simulation of the Burgers equation, a partial differential equation of importance in fluid mechanics [26]. However, unlike PCA/POD, our method does not attempt to reduce the *order* of the differential equations solved in our simulations. Rather, our method reduces the *number* of differential equations solved (by performing finite element analysis only in the regions of a microfluidic chip where it is absolutely necessary, the intersections).

In its current form, our instantaneous simulation method cannot be applied to arbitrary microfluidic chip designs. Rather, it is limited to chips with:

- “T” or “+”-shaped channel intersections
- 90° channel turns
- 200 *μ*m wide channels
- Fluid flow rates between 0 and 2 cm/s
- Particles with diameters between 1 and 20 *μ*m

However, there are several ways to extend the range of microfluidic devices that can be simulated instantaneously using our technique:

1. **In many cases, simply increasing the number and types of simulations in our pre-simulation database will allow our technique to apply to more microfluidic chip designs.** For example, our intersection simulation database currently contains only intersections with 90° angles between the intersecting channels. It would be fairly trivial to generate more pre-simulated intersections that cover a wider range of channel angles (perhaps every 30 degrees from 0 to 180 degrees). Generating these additional intersection simulations would take some computational time, but once they are added to the simulation database, they would not need to be generated again and could be immediately used to accelerate the simulation of chip designs with non-90° channel intersections.
2. **In some cases, a simulation in our database could apply to not just one but several different chip elements.** For example, at low Reynolds number (as is commonly encountered in microfluidics), the shape of turns in a channel have little effect on the locations of fluid streamlines and particle trajectories in the turn. In these cases, a wide range of channel turn angles could be accurately described by the 90° angle simulations already in our database (or the turn could even be modeled as an equivalent-length straight channel that our technique can model without using our database).
3. **In other cases, results from our database could be scaled to apply to other chip elements.** For example, our current database of channel intersections contains only 200 *μ*m wide channels. However, it is possible that these simulations could be scaled to apply to microfluidic chips with channel widths greater than or less than 200 *μ*m (as long as the new channel widths still provide for low-Reynolds-number, viscous-dominated flow). Using scaled simulation results would enable our technique to simulate a much wider range of channel dimensions.
4. **Many real-world microfluidic devices already adhere to the constraints of our technique and could therefore be simulated instantaneously using our current technique.** For example, sophisticated particle sorter chips have been demonstrated that utilize only 90° channel intersections [27]; these sorter chips could likely be simulated instantaneously using our current technique. Additionally, since the sizes of most human cells fall within the range of particle sizes already simulated in our current database (1 to 20 *μ*m), our technique should be suitable for simulating the behavior of many microfluidic cell sorters. Likewise, the randomly-designed microfluidic chips we developed previously (which are capable of generating any desired concentrations of a solute) consist entirely of “T” and “+”-shaped intersections and 90° turns [4], so they too can be simulated instantaneously using our technique. In summary, a wide range of useful microfluidic chips can already be simulated by our technique, even with its current limitations.
5. **Finally, if a researcher designs a microfluidic chip with the constraints of our technique in mind, they will be able to take advantage of instantaneous simulation.** In many situations, having a researcher limit their chip design to comply to our technique’s constraints would be a small price to pay for the accelerated simulation results that our technique provides. Naturally, not all microfluidic chips can be designed within these constraints, but for the chips that can be, doing so would guarantee that the designer can take advantage of instantaneous simulations throughout the design process.

## Supporting information

S1 File Raw data of simulation results from Figs 3 and 4. In MATLAB .*MAT* format (intended to be opened in MATLAB 2014 or later).
(ZIP)
